# Viral-host interaction in kidney reveals strategies to escape host immunity and persistently shed virus to the urine

**DOI:** 10.18632/oncotarget.14227

**Published:** 2016-12-26

**Authors:** Xumin Ou, Sai Mao, Yifan Jiang, Shengyong Zhang, Chen Ke, Guangpeng Ma, Anchun Cheng, Mingshu Wang, Dekang Zhu, Shun Chen, Renyong Jia, Mafeng Liu, Kunfeng Sun, Qiao Yang, Ying wu, Xiaoyue Chen

**Affiliations:** ^1^ Institute of Preventive Veterinary Medicine, Sichuan Agricultural University, Wenjiang, Chengdu City, Sichuan, People's Republic of China; ^2^ Key Laboratory of Animal Disease and Human Health of Sichuan Province, Sichuan Agricultural University, Wenjiang, Chengdu City, Sichuan, People's Republic of China; ^3^ Avian Disease Research Center, College of Veterinary Medicine, Sichuan Agricultural University, Wenjiang, Chengdu City, Sichuan, People's Republic of China; ^4^ China Rural Technology Development Center, Beijing, P.R. China

**Keywords:** duck Hepatitis A virus, virus-kidney interaction, viral distribution, kidney injury, comparative immunology, Pathology Section

## Abstract

Hepatitis A virus is one of five types of hepatotropic viruses that cause human liver disease. A similar liver disease is also identified in ducks caused by Duck Hepatitis A virus (DHAV). Notably, many types of hepatotropic viruses can be detected in urine. However, how those viruses enter into the urine is largely unexplored. To elucidate the potential mechanism, we used the avian hepatotropic virus to investigate replication strategies and immune responses in kidney until 280 days after infection. Immunohistochemistry and qPCR were used to detect viral distribution and copies in the kidney. Double staining of CD4+ or CD8+ T cells and virus and qPCR were used to investigate T cell immune responses and expression levels of cytokines. Histopathology was detected by standard HE staining. In this study, viruses were persistently located at scattered renal tubules. No CD4+ or CD8+ T cells were recruited to the kidney, which was only accompanied by transient cytokine storms. In conclusion, the extremely scattered infection was the viral strategy to escape host immunity and may persistently shed virus into urine. The deletion of Th or Tc cell responses and transient cytokine storms indeed provide an advantageous renal environment for their persistent survival.

## INTRODUCTION

Viral hepatitis caused by hepatitis viruses is not only associated with liver injury [1]. Kidney dysfunction is also a common complication caused by liver disease [1–3]. The filtration and excretion of the waste products of metabolism are the most important functions of the kidney. Interestingly, it has been reported that major hepatotropic viruses such as Hepatitis B virus (HBV), Hepatitis C virus (HCV), Hepatitis E virus (HEV) and Zika virus could be detected in urine, but they are not strictly limited to this classification [4–8]. This evidence indicated that it may be a universal strategy of the viral biological process for viruses to be released into urine. This result implied that viruses must transfer from blood or renal tissues to urine.

DHAV, a member of the Avihepatovirus (Avian Hepatovirus) genus in the family Picornaviridae, causes a highly fatal, rapidly infectious disease in ducklings that is characterized by swelling livers mottled with hemorrhages [9, 10]. However, their replication strategies and long-term survival caused by hepatotropic virus in kidney have not been fully revealed [4]. The basic pathogenesis is extremely complex and is involved in the impaired energetics of nephrons that are caused by tubular damage, glomerular damage, and interstitial damage [11]. Immune inhibition caused by viral non-structural proteins and excessive activation of immune responses may be the explicable factors contributing to persistent survival in kidney [11–13]. The activation and recruitment of immune cells into injured kidneys may be universal strategies to protect against infection. However, the recruitment of neutrophils, dendritic cells, macrophages and lymphocytes also contributes to the pathogenesis and repair of renal injury [14]. In the course of ischemic acute renal failure, CD4+ T cells but not CD8+ T cells were a major pathogenic factor for severe tubular damage [15]. Additionally, the strong elevation of cytokines secreted by those immune cells and activated pattern recognition receptors are a double-edged sword because of their therapeutic effect and pathological injury [14, 16].

Recently, it has been reported that three types of hepatotropic viruses (HBV, HCV, and HEV) can persistently shed virus into urine [4–6]. It seems that hepatotropic viruses may be released from renal tubules or glomeruli and then shed virus to the urine. However, how the viruses escape host immunity and persistently enter into the urine has not been fully explored. To elucidate the potential mechanism, we used an avian hepatotropic virus (DHAV) to investigate its replicated characteristics and viral induced immune responses in kidney.

## RESULTS

### DHAV was persistently distributed at scattered renal tubules during long-term infection

**Figure 1:**
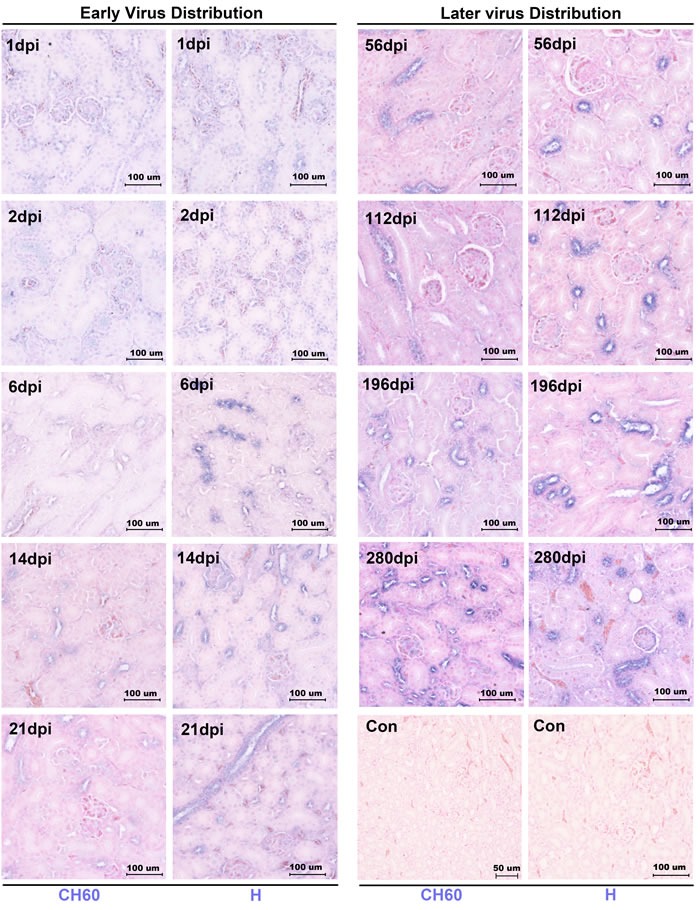
Dynamic viral distribution in kidney infected with the DHAV-CH60 strain and H strain Representative virus distributions were detected by immunohistochemical assay, and sections were counterstained with nuclear fast red (*n* = 5). Viral distribution is displayed as blue-violet. The early viral distribution and later viral distribution are shown on the left and right, and the kidneys infected with H strain or CH60 strain are also labeled at the bottom of the figure. Notably, virus was persistently distributed at scattered renal tubules until 280 dpi. The brightness and contrast are slightly modified to create a uniform background.

In this study, we identified that DHAV was mainly located at mesangial cells and vascular endothelial cells at the early stage of infection. Both strains of DHAV, a virulent strain (H strain) and an attenuated strain (CH60 strain), could scatter, infect and replicate at the renal tubules in the very long-term, especially in the distal tubules and collecting tubules (Figure 1). Two strains of DHAV could transmit from inoculated sites to the blood capillary at the early stage of infection. Then, they would persistently locate at renal tubules until 280 days after infection (dpi) (Figure 1).

### Renal tubules and glomerulus lesions were associated with kidney injury

**Figure 2:**
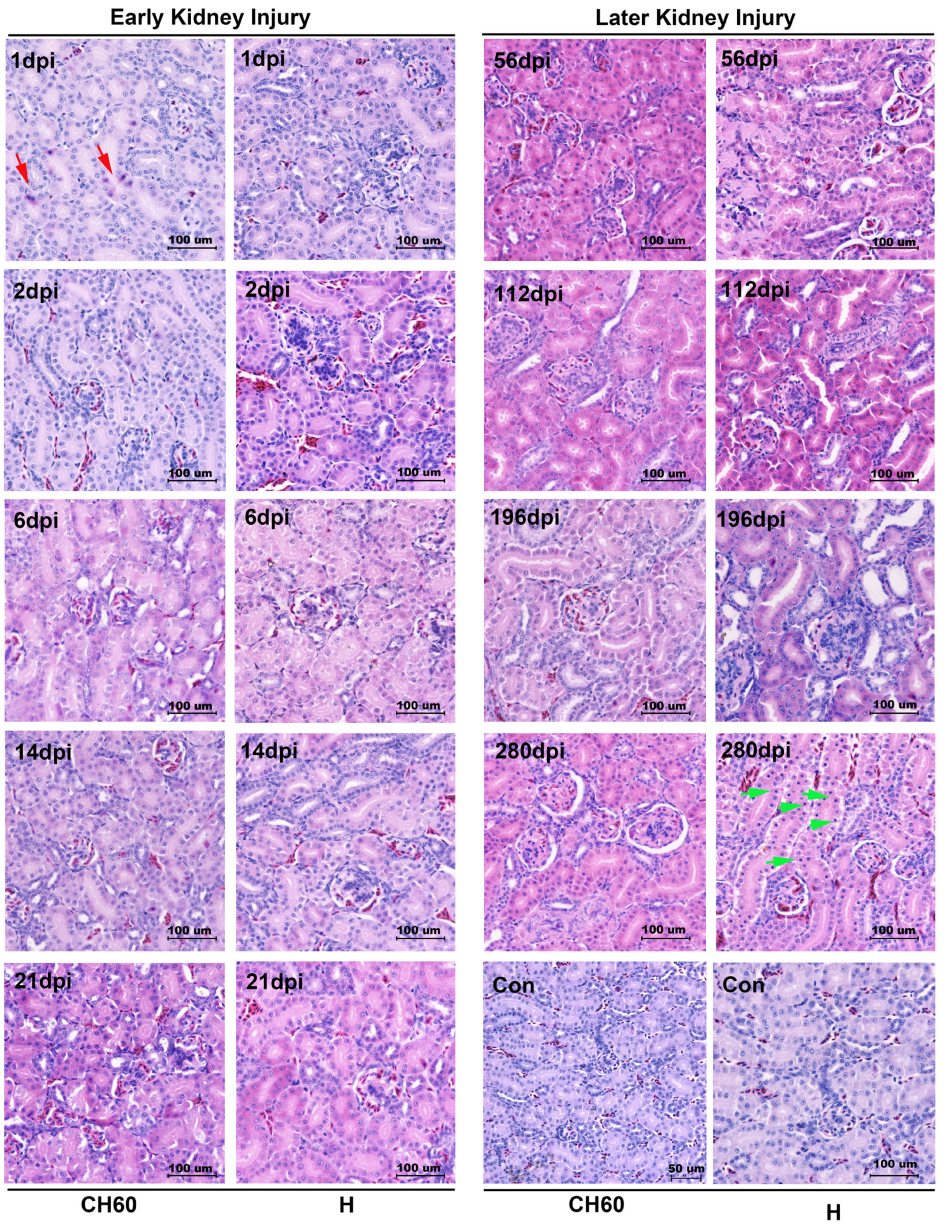
Microscopic lesions in kidney infected with the DHAV-1 H strain and CH60 strain Representative histopathological changes were detected by standard HE Staining (*n* = 5). Representative instances of cellular apoptosis and necrosis are indicated as red arrows and green arrows, respectively. Glomerulonephritis were also identified, especially in the later stage of infection. The brightness and contrast are slightly modified to create a uniform background.

Cellular apoptosis and necrosis were the main consequence of cell death caused by infection or other pathological factors. In the initial stage of infection, renal tubule lesions were characterized by scattered cellular apoptosis, swelling or granular degeneration (Figure 2). In the later stage of infection, kidney lesions were characterized by cell necrosis, especially in virulent strain-infected kidneys at 280 dpi (Figure 2). Additionally, the lesion of glomerulus was characterized by glomerulonephritis, especially in the later stage of infection. The strength of the injury caused by attenuated virus is relatively lower than that caused by virulent virus. Additionally, none of neutrophil granulocytes were recruited to the kidney (Figure 2).

### No CD4+ or CD8+ T cells was recruited to the kidney to protect against viral infection

**Figure 3:**
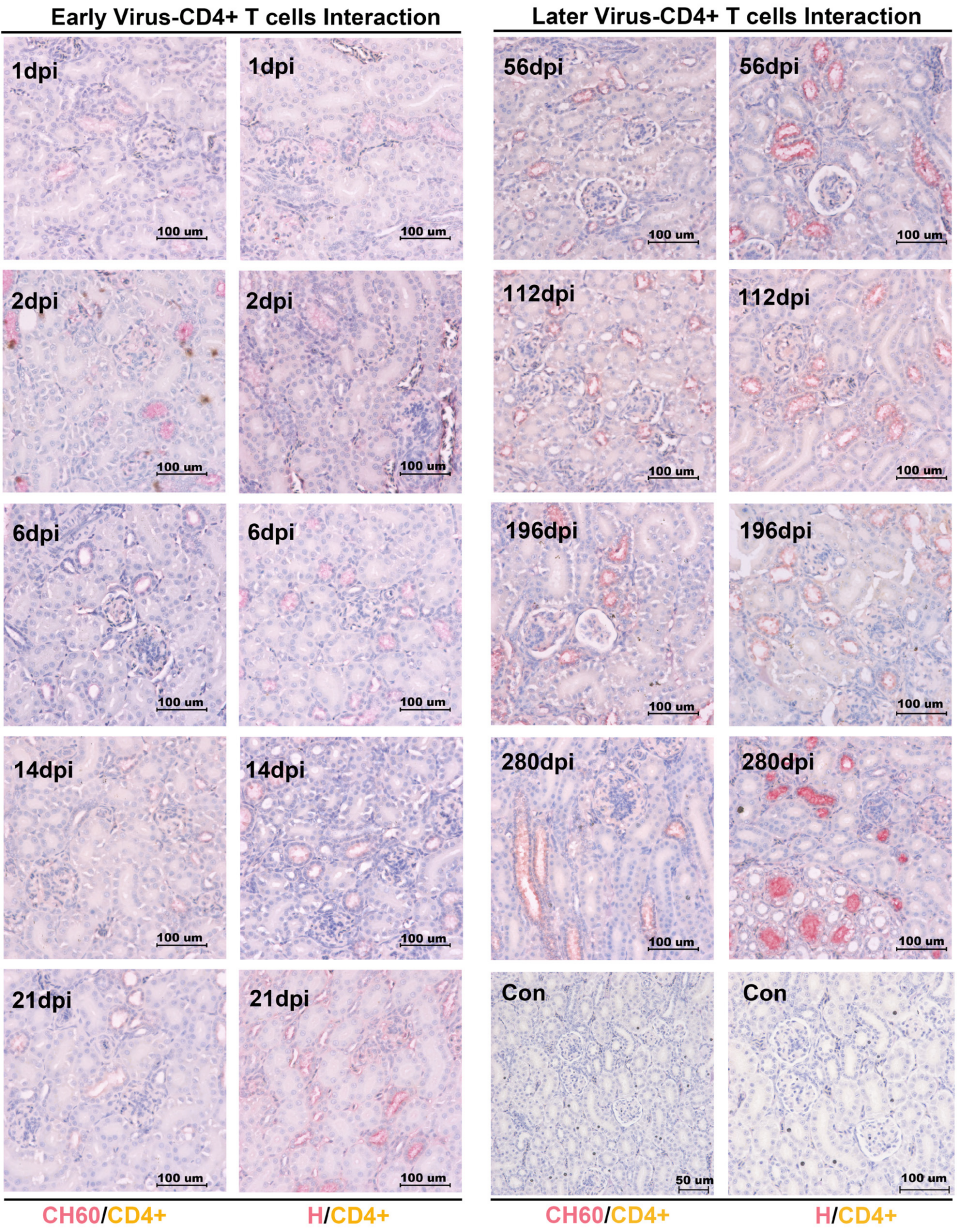
Double staining of viral capsid and CD4+ positive T Cells in kidney infected with the DHAV-CH60 strain and H strain Viral capsid and CD4+ T cells were double stained by rabbit anti-DHAV polyclonal antibody and mouse anti-duck CD4+ monoclonal antibody (AbD Serotec MCA2478) (*n* = 5). Red color and brown color represent positive capsid antigens and CD4+ T cells, respectively. (Con) represents kidney without double primary antibody. The brightness and contrast are slightly modified to create a uniform background.

To investigate the viral clearance caused by CD4+ or CD8+ T cells, the double staining of the vial capsid and CD4+ or CD8+ T cells was used to address this question. However, no positive staining of CD4+ or CD8+ T cells from 1 dpi to 280 dpi was identified in our study (Figures 3/4). Occasionally, the positive staining of CD8+ T cells was only identified in CH60 strain-infected kidneys at 8 dpi.

**Figure 4:**
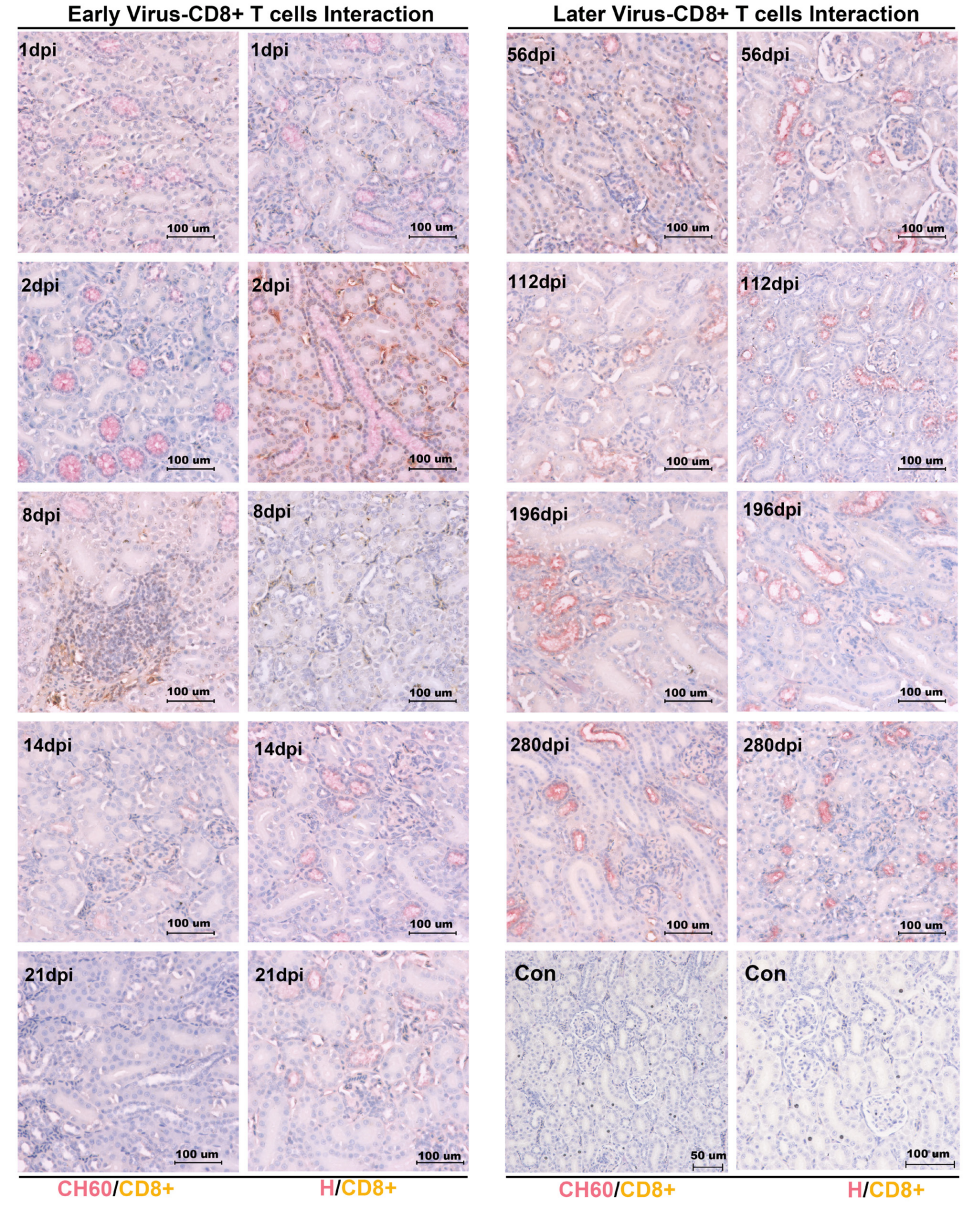
Double staining of viral capsid and CD8+ positive T Cells in kidney infected with the DHAV-CH60 strain and H strain Viral capsid and CD8+ T cells were double stained by rabbit anti-DHAV polyclonal antibody and mouse anti-duck CD8+ monoclonal antibody (AbD Serotec MCA2479) (*n* = 5). Red color and brown color represent positive capsid antigens and CD8+ T cells, respectively. (Con) represents kidney without double primary antibody. The brightness and contrast are slightly modified to create a uniform background.

### Cytokine storm was negatively related to virus replication but positively related to acute kidney injury

**Figure 5:**
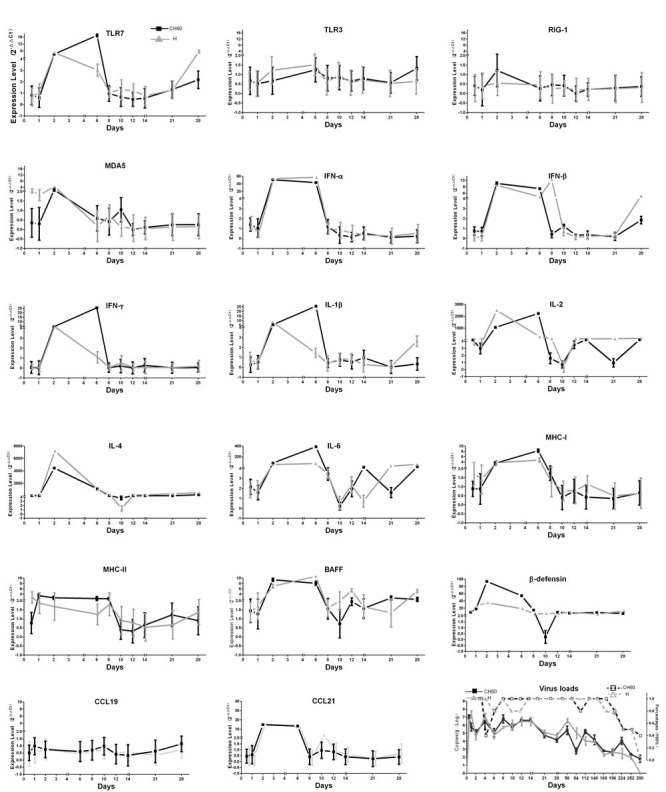
Dynamic changes of immune-related genes and virus loads in kidney infected with the DHAV-1 CH60 strain and H strain Immune-related genes at different stages of immune responses were selected, such as the activation of innate immune responses (TLR7, TLR3, RIG-1 and MDA5), effective interferons (IFN-α/β/γ) and interleukins (IL-1β/2/4/6), chemokines (CCL19/21), MHC-I and MHC-II, β-defensin, and B cell activating factor (BAFF). Most immune-related genes in both the CH60 strain and H strain-infected kidneys were up-regulated from 2 dpi to 6 dpi. Those cytokine storms were related to a rapid decrease of virus replication in the kidney. The virus loads in kidney infected with the DHAV-CH60 strain and H strain were plotted at the lower right corner, and their percentage in each group is also shown (*n* = 5).

To clearly elucidate the early immune responses, immune-related genes at different stages were selected, such as innate immune responses (Toll-like Receptor (TLR7), TLR3, Retinoic acid-inducible gene-1 (RIG-1) and Melanoma Differentiation-Associated protein 5 (MDA5)), effective interferons (IFN-α/β/γ) and interleukins (IL-1β/2/4/6), chemokines (CCL19/21), Major Histocompatibility Complex class I (MHC-I) and MHC-II, β-defensin, and B cell activating factor (BAFF). In this study, most immune-related genes in both the CH60 strain and H strain-infected kidneys were up-regulated from 2 dpi to 6 dpi (Figure 5). The cytokine storm, interferon (IFN-α/β/γ) and interleukins (IL-1β/2/4/6) were related to a rapid decrease of virus replication in the kidney (Figure 5). Meanwhile, obvious renal injury identified at 6 dpi was also coincident with a strong cytokine storm, and this result indicated that cytokines may have some pathological effect on renal tubules (Figures 2/5). During the early infection, TLR7 and MDA5 were also highly up-regulated from 2 dpi to 6 dpi in both virulent and attenuated strain-infected kidneys. The changes in MHC-I, MHC-II and CCL21 were coordinated with those interferon and interleukins. The expression levels of CCL19 were constant during all stages of infection. Additionally, BAFF and β-defensin were also highly up-regulated. Due to the diversity of the virulence of the CH60 strain and H strain, the expression levels of some cytokines induced by those two strains were different. In our study, IFN-γ, IL-1β/4/6 and β-defensin were identified as the main differentially regulated genes induced by a diversity of virulence (Figure 5).

### Virulence may shape the immune networks in the early stage of infection

**Figure 6:**
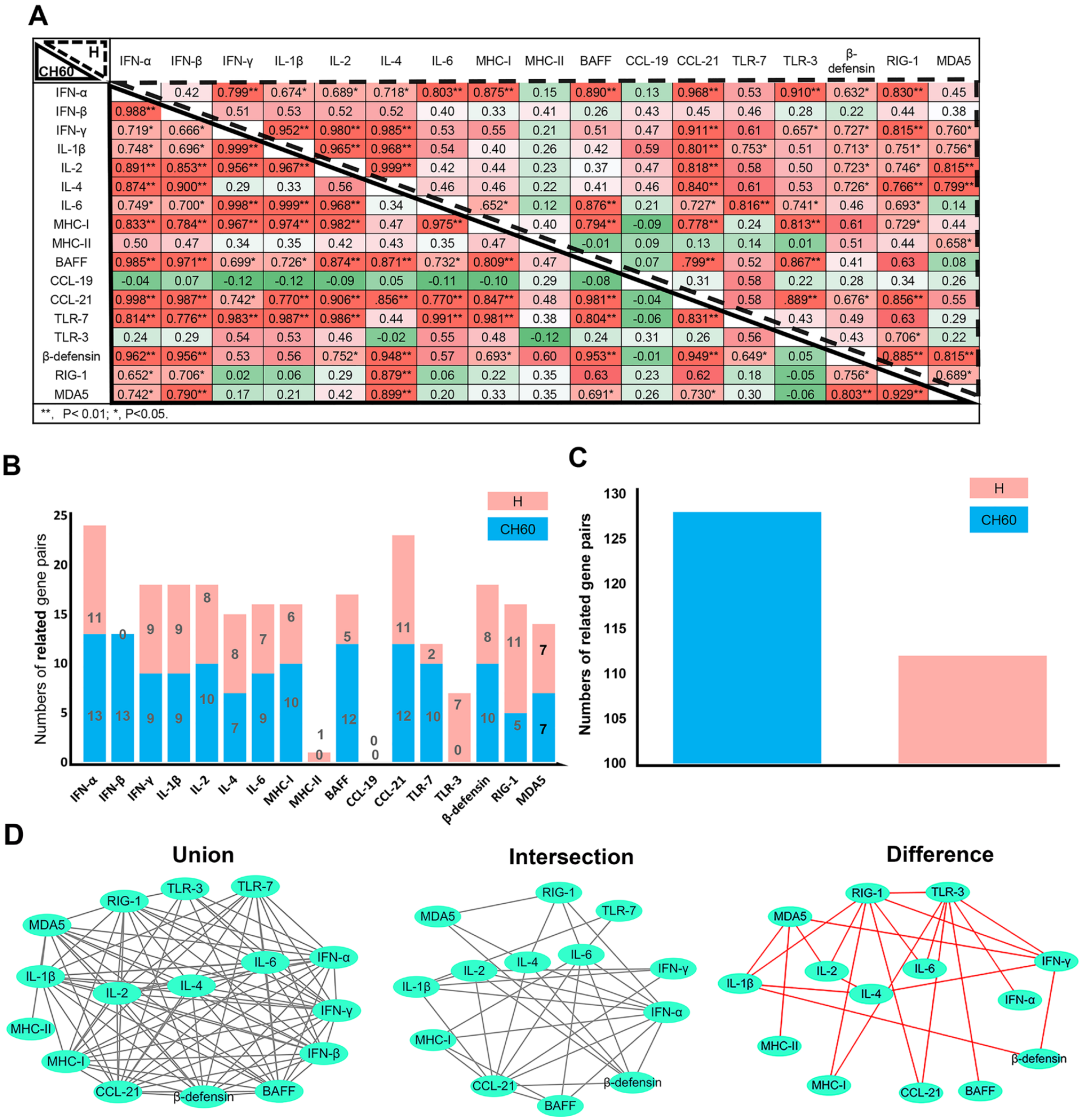
Immune networks induced by the DHAV-1 H strain and CH60 strain ( ***n*** = 5). To understand the impact of virulence on immune networks, the correlations of each pair of immune-related genes (*P* < 0.05 at least) were calculated using correlation analysis (Pearson). **A**. Correlation matrices of immune-related genes in kidney infection with the H strain and CH60 strain are displayed at the left bottom corner and top right corner, respectively. The values represented the strength of the correlation. *, *P* < 0.05; **, *P* < 0.01. **B**. Comparative analysis of correlations between those immune-related genes indicated that IFN-β and TLR3 were the differently regulated genes induced by the CH60 strain and H strain. **C**. The total number of related gene pairs in the course of CH60 strain-infected kidneys were significantly higher than for H strain-infected kidney. **D**. Those correlated pairs of immune-related genes were visualized by the Cytoscape software. The union, intersection and difference of those pairs were also analyzed and displayed to compare the immune networks shaped by the CH60 strain and H strain. The changes of MHC-II and CCL19 were not related to any other immune genes except for MDA5 and MHC-II in H strain-infected kidney. The differences of those pairs were identified in cytokine storms caused by the H strain. Red color represents the specific pairs caused by the H strain.

To understand the impact of virulence on immune networks, the correlations of each pair of immune-related genes were calculated using correlation analysis (Pearson). The total number of related gene pairs in the course of CH60 strain-infected kidneys was significantly higher than that of H strain-infected kidneys (Figure 6A, 6C). A comparative analysis of those immune-related genes indicated that IFN-β and TLR3 were the top two differently regulated genes induced by the CH60 strain and H strain (Figure 6B). The changes of MHC-II and CCL19 were not related to any other immune genes except for MHC-II and MDA5 in the H strain-infected kidney (Figure 6B, 6D). To further understand those immune networks, the intersections and differences of the networks were also identified. IFN-α, CCL21, β-defensin and IL-1β/2/4/6 were the high frequency genes correlated with each other in both strains of infected kidney. MDA5, RIG-1, TLR3 and IFN-γ were the top four differently regulated genes induced by the H strain (Figure 6D).

## DISCUSSION

**Figure 7:**
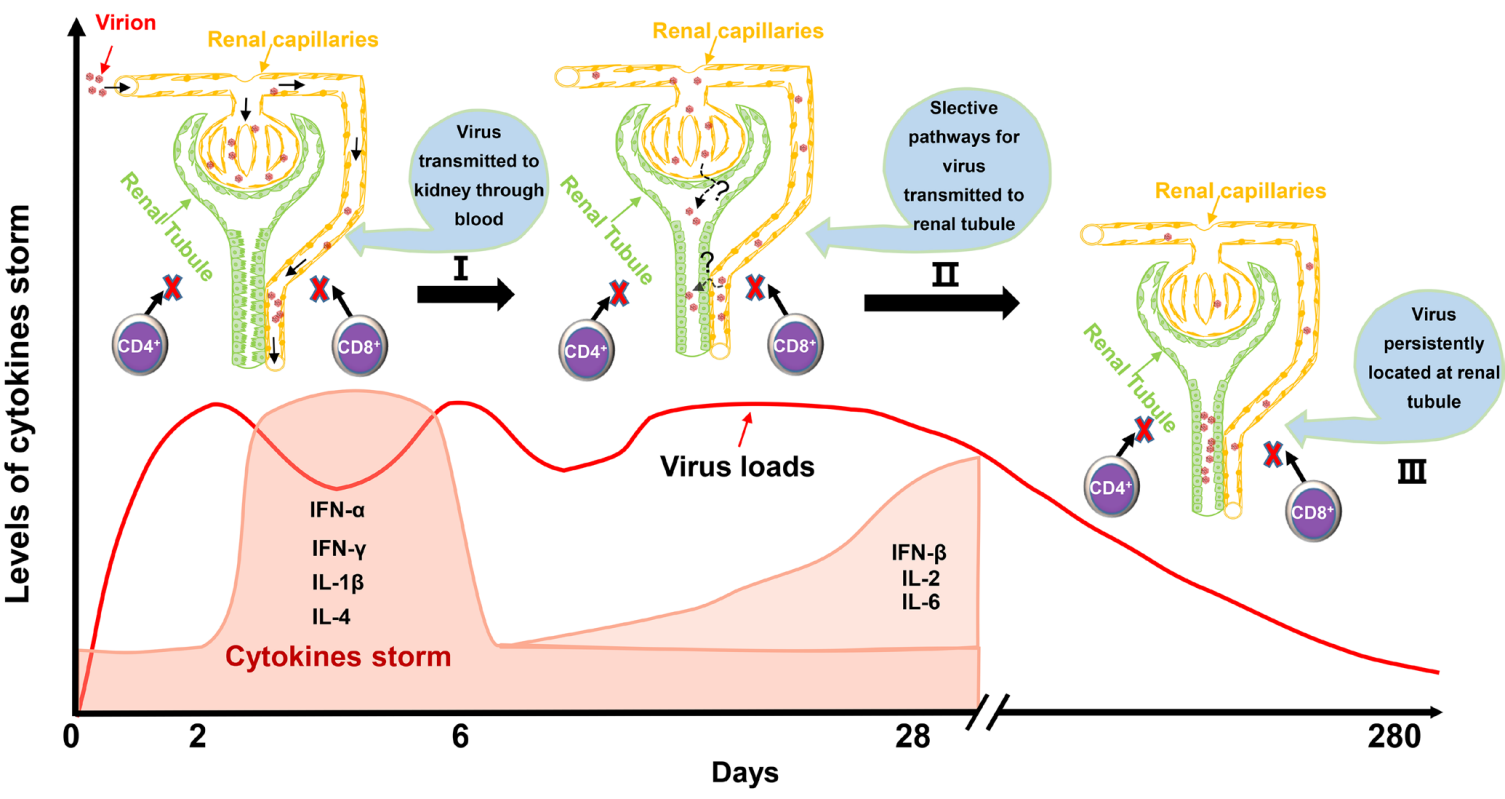
Schematic of virus-kidney interaction during the early stage and later stage of infection Mesangial cells and vascular endothelial cells were the main identified cells for early infection. Then, a strong cytokine storm from 2 dpi to 6 dpi was induced that was accompanied by a sudden decrease of virus loads in the kidney. Scattered renal tubules were the main target of cells for later infection until 280 dpi. It should be mentioned that no CD4+ T cells or CD8+ T cells were recruited to the kidney for viral clearance during the entire period of infection. Those characteristics of the virus-kidney interaction would provide basic and valuable information to elucidate persistent infection and virus shedding from kidney to urine.

In this study, we identified that DHAV was mainly located at mesangial cells and vascular endothelial cells at the early stage of infection and progressed to scattered infection of renal tubules at the later stage of infection. The extremely scattered infection was the viral strategy for long-term survival in renal tissues, because overall infection was lethal to their host [17]. Notably, the biomacromolecules, such as antibodies, were not permitted to filtrate from the glomerulus to crude urine. For the virus, a lack of antibodies in the renal tubules may also allow them to escape humoral immunity. Additionally, viral replication indeed has some pathological injury. The histopathological observation indicated that cellular apoptosis and necrosis were the main consequences of DHAV infection, especially in virulent strain-infected kidney (Figure 2). However, neither CD4+ nor CD8+ T cells were detected during the whole period of infection and neither were lymphocytes [18, 19]. Those results suggested that virus could persistently infect scattered renal tubules without the recruitment of immune cells (Figure 7). This deletion may provide an advantageous renal environment for persistent survival.

To our understanding, hepatotropic virus can infect many vertebrates such as humans, mice, pigs, bats and birds [20–23]. DHAV is indeed an avian hepatotropic virus that is classified into the Picornavirus family with human Hepatitis A virus (HAV) [10]. Duck viral hepatitis caused by DHAV is a highly fatal, rapidly infectious disease in ducklings [10]. In contrast, human HAV caused acute hepatitis in both teenagers and adults. Notably, both DHAV and HAV have a fecal-oral transmission route. Phylogenetic analysis indicated that the DHAV members of Avihepatovius were very close to the human HAV, and both belong to Picornaviridae [24]. Due to those similar characteristics, we predict that the other hepatotropic viruses may utilize analogous strategies to persistently survive in the kidney and shed virus to urine [8].

Cytokines, interferons and interleukins are vital for both antiviral responses and the pathogenesis of viral infection. In this study, a strong cytokine storm from 2 dpi to 6 dpi was induced by both virulent and attenuated strains, including type I (IFN-α/β) and type II IFNs (IFN-γ), Th1-related ILs (IL-1β/2/6) and Th2-related ILs (IL-4). This storm was consistent with both viral decrease and kidney injury (Figure 2/5). Combined with the histopathological changes, those results indicated that cytokine storm was a double-edged sword to the host because they not only enhanced viral clearance but also had a pathogenic effect [25]. The excessive elevation of cytokine storms did indeed cause pathological injury at early viral infection (Figure 2) [17, 26, 27]. This result is mainly caused by the activation of Toll-like receptors, MDA5 or RIG-1-mediated pathways [26, 28, 29]. In our study, the strong elevation of TLR7 may be involved in early kidney injury. These results are similar to prior results showing that the activation of TLR7 in plasmacytoid dendritic cells will lead to glomerulonephritis [30]. Additionally, type I interferon activated by MDA5 in human mesangial cells was also involved in renal injury [31]. Similar to this finding, MDA5 was highly up-regulated and correlated with IFN-α (*P* < 0.05) [32] (Figure 5/6). In contrast, the virulent and attenuated strains induced different correlations between cytokines (Figure 6A, 6B, 6C, 6D). This difference might be caused by genetic variations during a series of passaging in chicken embryos because some of those mutations are directly correlated with their virulence.

Accompanying the cytokine storm, the other immune-related genes were also involved in this storm, such as MHC-I, MHC-II, CCL21, BAFF and β-defensin. MHC-I and MHC-II, expressed on the surface of antigen-presenting cells (APCs), are responsible for activating CD8+ cytotoxic or CD4+ T helper cells, respectively [18, 33, 34]. Chemokines were bound for leukocyte trafficking during the infection [35]. BAFF was vital for the differentiation and proliferation of B cells [36]. However, none of the T cells (Tc or Th cells), B cells or leukocytes were recruited to the kidney. The exact underlying reasons will need further investigations. Additionally, β-defensin has been shown to be an anti-inflammatory cytokine [37, 38]. In this study, β-defensin persistently up-regulated in kidney may support long-term survival, which is similar to the fact that influenza virus can persistently survive in reservoir host-ducks with a strong elevation of β-defensin [39].

In summary, we identified that DHAV could persistently locate at scattered renal tubules. None of the T helper or cytotoxic T cells were recruited to the kidney, which only accompanied transient cytokine storms (Figure 7) [17]. Those viral strategies are vital to escape T cell and humoral immunity and persistently survive and shed virus into urine. Those findings are significantly important for understanding viral-host interactions and have important implications for viral biological processes.

## MATERIALS AND METHODS

### Ethics statement

The 160-day-old breeding ducks were purchased at Mianying company (http://www.mianying.com), and this study was performed in strict accordance with the recommendations in the ARRIVE guidelines (http://www.nc3rs.org.uk/arrive-guidelines). The animal experiments were approved by the committee of experiment operational guidelines and animal welfare of Sichuan Agricultural University, China (the approved permit number is XF2014-18). All ducks were handled in compliance with animal welfare regulations and maintained according to standard protocols. All surgeries were performed on animals under sodium pentobarbital anesthesia, and all efforts were made to minimize suffering.

### Virus strains

The DHAV-1 CH60-attenuated vaccine (GenBank: KU923754.1) and a DHAV-1 H strain (GenBank: JQ301467.1) were selected to investigate their replication and differential effects on immune responses. The attenuated strain, which was derived from the DHAV-1 CH strain in the allantoic cavities of 9-day-old specific pathogen-free (SPF) chicken embryos after 60 passages, is a commercial vaccine developed by our laboratory. The DHAV-H strain was propagated in 9- to 11-day-old duck embryos by standard procedures. The embryos died at 36-72 hpi and were harvested. The homogenates of chicken embryonic bodies and the allantoic fluids from the duck embryos were stored at -80°C until use. Virus at a concentration of 4.56 × 10^8^ copies/ml as determined by quantitative real-time PCR (qPCR) was used to infect ducks.

### Experimental design

In total, 205 ducks were randomly divided into 41 groups. Group 1 to group 20 received 1 ml of DHAV-H strain (4.56 × 10^8^ copies/ml) by intramuscular injection, group 21 to group 40 received 1 ml of DHAV-CH60 strain (4.56 × 10^8^ copies/ml) by intramuscular injection, and group 41 was injected with an equal volume of 0.85% physiological saline as a negative control. The kidneys of the 41 experimental groups were collected according the time post infection, including 0.5 d, 1 d, 2 d, 4 d, 6 d, 8 d, 10 d, 12 d, 14 d, 21 d, 28 d, 56 d, 84 d, 112 d, 140 d, 168 d, 196 d, 224 d, 252 d, and 280 d. One hundred milligrams of kidney specimens were weighed and then immediately cryopreserved in liquid nitrogen until being processed for RNA isolation. The adjacent tissues were used for histopathological examination and immunohistochemistry.

### HE staining and immunohistochemistry

After administering sodium pentobarbital anesthesia, the kidneys from the same samples used for transcriptional analysis were fixed in 4% paraformaldehyde, dehydrated, embedded in paraffin, sectioned into 4-μm thick sections and stained with hematoxylin and eosin (HE) using standard procedures. Paraffin-embedded kidney tissues were deparaffinized in xylene and rehydrated in graded alcohols. For antigen retrieval, slides were boiled in Tris/EDTA pH 9.0 for 20 min. Then, 0.01 M HCl was used to block endogenous alkaline phosphatase for 15 min at room temperature (RT). Then, 3% H_2_O_2_ was used to block endogenous peroxidase for 15 min at RT. The slides were incubated in 5% BSA blocking solution followed by overnight incubation at 4°C in rabbit anti-DHAV polyclonal antibody (1:20 dilutions). Alkaline phosphatase conjugated goat anti-rabbit IgG (1:1000 dilutions, Life Technology) was incubated for 30 min at 37°C. The positive staining was colored with BCIP/NBT solution for 20 min at RT and counterstained with nuclear fast red for 20 min at RT. Additionally, double staining of DHAV and CD4+ or CD8+ T cells was first incubated with a cocktail of rabbit anti-DHAV polyclonal antibody and mouse anti-duck CD4+ or CD8+ monoclonal antibody (1:20 dilutions and 1:200 or 1:100 dilutions, respectively) followed by a mixture of HRP coupled goat anti-rabbit and alkaline phosphatase coupled donkey anti-mouse secondary antibody mixture. Then, positive staining was colored with DAB solution for 10 min and permanent red solution for 15 min at RT and counterstained with hematoxylin.

### RNA isolation and cDNA preparation

Total cellular RNA was isolated from 100 mg of renal tissue using the RNAiso plus Reagent (TaKaRa, Japan) according to the manufacturer's protocols. The RNA isolated from each specimen needed to detect immune-related genes was treated with 1.0 µl of gDNA Eraser (Perfect Real Time, Janpan) for two min at 42°C to remove the potential contaminating genomic DNA (gDNA) and then used to carry out reverse transcription to produce cDNA with the PrimeScriptTM RT Reagent Kit according to the manufacturer's instructions (TaKaRa, Janpan).

### qPCR

Viral copies were detected by previously established methods in our laboratory [40]. Seventeen immune-related genes (IL-1β, IL-2, IL-4, IL-6, IFN-α, IFN-β, IFN-γ, MHC-I, MHC-II, CCL19, CCL-21, BAFF, TLR3, TLR7, β-defensin, RIG-1 and MDA5) and a housekeeping gene (glyceraldehyde-3-phosphate dehydrogenase (GAPDH)) were detected by qPCR. The primer sequences for detecting the immune gene transcripts were previously published [41] (Table S1). The primer sequences for IL-4, BAFF, CCL19, CCL21, TLR3, β-defensin, RIG-1 and MDA5 were newly designed in this study using Primer 3 input version 0.4.0 (http://bioinfo.ut.ee/primer3-0.4.0/). All primers used in this study are shown in Table S1. The expression levels of mRNA transcripts were determined by qPCR using the SYBR^®^Premix Ex Taq™ II (Tli RNaseH Plus) Kit (Takara). The amplification procedure was performed in a 20 µl reaction volume containing 8 µM of each primer and 2 µl of RNA. The following thermal cycling conditions were used: PCR initial activation at 95°C for 30 s, 45 cycles of denaturation at 95°C at 5 s and annealing and extension at 58.2°C for 30 s.

### qPCR and statistical analyses

Relative gene expression data were analyzed using the 2-ΔΔCt method by comparing with the control group injected with 1 ml normal saline (NS) [42], and ΔCt values were determined by subtracting the average Ct values of the endogenous control gene GAPDH from the average Ct values of target genes. The photographs were generated using GraphPad Prism 5 software. Standard Pearson's correlation coefficients were determined using fold changes of immune-related genes in kidney with the SPSS 20.0 statistical software. Those correlated pairs with significant correlations were visualized by Cytoscape software.

## SUPPLEMENTARY MATERIAL TABLES FIGURE



## Abbreviations

HAV: Hepatitis A virus
DHAV: Duck Hepatitis A virus
HBV: Hepatitis B virus
HCV: Hepatitis C virus
HEV: Hepatitis E virus
qPCR: quantitative real-time PCR
Tc: cytotoxic T cell
Th: helper T-cell
dpi: day(s) post infection
