# Supplementary material for: Viral-host interaction in kidney reveals strategies to escape host immunity and persistently shed virus to the urine
Source: Oncotarget. 2016 Dec 26;8(5):7336–49. doi: 10.18632/oncotarget.14227 (PMC5352325; doi:10.18632/oncotarget.14227)
Supplement: Supplementary file 1 [file oncotarget-08-7336-s001.pdf]

Viral-host interaction in kidney reveals strategies to escape host immunity and persistently shed virus to the urine

Supplementary Material

Table of contents

Supplementary materials and methods……………………………………….2

qPCR data of kidney induced by CH60 strain ……………………………….4

qPCR data of kidney induced by H strain…………………………………….5

Data matrix of Pearson Correlation Coefficient caused by CH60 and H strains……………………………………………………………………………….6

References………………………………………………………………………7

Supplementary materials and methods

qPCR

Seventeen immune-related genes (IL-1β, IL-2, IL-4, IL-6, IFN-α, IFN-β, IFN-γ, MHC-I, MHC-II, CCL19, CCL-21, BAFF, TLR3, TLR7, β-defensin, RIG-1 and MDA5) and a housekeeping gene glyceraldehyde-3-phosphate dehydrogenase (GAPDH) were detected by qPCR. Primer sequences for detecting the immune gene transcripts were previously published [1] (Tab 2). The primer sequences for IL-4, BAFF, CCL19, CCL21, TLR3, β-defensin, RIG-1 and MDA5 were newly designed in this study using Primer 3 input version 0.4.0 (http://bioinfo.ut.ee/primer3-0.4.0/), and non-specific products were eliminated by using online NCBI BLAST searches. All primers used in this study are shown in S1Table. Expression levels of mRNA transcripts were determined by qPCR using the SYBR®Premix Ex Taq™ II (Tli RNaseH Plus) Kit (Takara). The amplification procedure was performed in a 20 μL reaction volume containing 8 μM of each primer and 2 μl of RNA. The following thermal cycling conditions were used: PCR initial activation at 95°C for 30 s, 45 cycles of denaturation at 95°C at 5 s, annealing and extension at 58.2°C for 30 s.

Table 1 primer sequences used in gene expression profiles

Gene	Forward primer	Reverse primer	AD	Reference
GAPDH	5’-ATGTTCGTGATGGGTGTGAA-3’	5’-CTGTCTTCGTGTGTGGCTGT-3’	AY436595	[38]
IFN-α	5’-TCCTCCAACACCTCTTCGAC-3’	5’-GGGCTGTAGGTGTGGTTCTG-3’	EF053034	[38]
IFN-β	5’-CCTCAACCAGATCCAGCATT-3’	5’- GGATGAGGCTGTGAGAGGAG-3’	AY831397	[38]
IFN-γ	5’-GCTGATGGCAATCCTGTTTT-3’	5’- GGATTTTCAAGCCAGTCAGC-3’	AJ012254	[38]
IL-1β	5’-TCGACATCAACCAGAAGTGC-3’	5’-GAGCTTGTAGCCCTTGATGC-3’	DQ393268	[38]
IL-2	5’-GCCAAGAGCTGACCAACTTC-3’	5’-ATCGCCCACACTAAGAGCAT-3’	AF294323	[38]
IL-4	5’-CCTCCACGGTTGTTTTCGAG-3’	5’-GTTGGAGGGTTCTGTGGAGG-3’	XM_005024359.1	new
IL-6	5’-TTCGACGAGGAGAAATGCTT-3’	5’-CCTTATCGTCGTTGCCAGAT-3’	AB191038	[38]
MHC-I	5’-GAAGGAAGAGACTTCATTGCCTTGG-3’	5’-CTCTCCTCTCCAGTACGTCCTTCC-3’	AB115246	[38]
MHC-II	5’-CCACCTTTACCAGCTTCGAG-3’	5’-CCGTTCTTCATCCAGGTGAT-3’	AY905539	[38]
BAFF	5’-TGTGCACGTCATCCAACAGA-3’	5’-GCCACAGGAATGTGACAGGA-3’	DQ445092	new
CCL21	5’-GGAGAAGCAGAAGAACCCCC-3’	5’-GGGAAAGCATCCGTCCTCTC-3’	DR764376	new
CCL19	5’-CCAGGAAGGTCCCAAATAAA-3’	5’-GTAGTAGGAGGTGGAAGCAAGTC-3’	DR766004	new
β-defensin	5’-CCAGGTTTCTCCAGGATTGT-3’	5’-AACCCAAAGCAACTTCCAAC-3’	AY641439	new
TLR7	5’-CCTTTCCCAGAGAGCATTCA-3’	5’-TCAAGAAATATCAAGATAATCACATCA -3’	AY940195	[38]
TLR3	5’-AACACTCCGCCTAAGTATCAT-3’	5’-CTATCCTCCACCCTTCAAAA-3’	JN573268	new
RIG-1	5’-GCGTACCGCTATAACCCACA-3’	5’-CCTTGCTGGTTTTGAACGC-3’	AB772012.1	new
MDA5	5’-GCTGAAGAAGGCCTGGACAT-3’	5’-TCCTCTGGACACGCTGAATG-3’	KJ451070.1	new

Real-time PCR and statistical analyses

Relative gene expression data were analyzed using the 2-ΔΔCt method by comparing with the control group injected with 1 ml normal saline (NS) [18], and ΔCt values were determined by subtracting average Ct values of the endogenous control gene GAPDH from average Ct values of target genes. The photographs were generated using GraphPad Prism 5 software. Standard Pearson’s correlation coefficients were determined using fold changes of immune related genes in kidney with SPSS 20.0 statistical software. Those correlated pairs with significant correlation were visualized by Cytoscape software.

qPCR data of kidney induced by CH60 strain

Table 1 Fold changes of immune related genes induced by DHAV-CH60 strain ($2^{-\Delta\Delta Ct}$)

CH60	IFN-α	IFN-β	IFN-γ	IL-1β	IL-2	IL-4
0.5d	1.41±0.66	0.76±0.43	0.06±0.59	0.26±0.78	6.37±0.62	6.83±0.5
1d	1.06±0.94	0.7±0.43	0.03±0.7	0.51±0.67	2.99±0.76	7.03±0.67
2d	50.83±0.89	10.64±0.68	4.29±0.51	5.66±0.54	1064.95±0.48	4462.75±0.54
6d	43.26±0.86	8.17±0.13	25.79±0.61	25.65±0.61	2235.79±0.84	1109.13±0.59
8d	1.2±0.72	0.43±0.34	0.05±0.47	0.43±0.44	1.61±0.76	5.82±0.51
10d	0.36±0.83	1.25±0.19	0.2±0.67	0.75±0.65	0.73±0.49	3.77±0.44
12d	0.15±0.47	0.36±0.12	0.02±0.6	0.53±0.7	3.48±0.53	11.5±0.65
14d	0.52±0.65	0.37±0.4	0.27±0.69	0.96±0.76	13.55±0.44	11.06±0.47
21d	0.12±0.56	0.19±0.38	0.03±0.56	0.02±0.64	0.96±0.59	4.3±0.49
28d	0.24±0.65	1.86±0.38	0.05±0.46	0.34±0.63	25.06±0.57	174.09±0.64
CH60	IL-6	MHC-I	MHC-II	BAFF	CCL-19	CCL-21
0.5d	2.17±0.77	0.89±0.42	0.77±0.59	1.45±0.73	0.51±0.55	0.46±0.59
1d	1.59±0.74	0.88±0.85	3.43±0.78	1.27±0.83	0.98±0.6	0.59±0.78
2d	83.49±0.57	3.52±0.63	2.64±0.62	9.41±0.62	0.75±0.58	14.86±0.73
6d	387.35±0.77	8.29±0.6	2.35±0.79	8.07±0.77	0.6±0.71	13.46±0.7
8d	3.46±0.63	1.75±0.82	2.28±0.52	1.56±0.8	0.7±0.69	0.42±0.66
10d	0.22±0.39	0.41±0.67	0.4±0.53	0.73±0.78	0.98±0.62	0.96±0.69
12d	1.97±0.79	0.76±0.66	0.32±0.65	1.95±0.66	0.41±0.68	0.83±0.66
14d	5.37±0.45	0.43±0.66	0.65±0.69	1.58±0.65	0.33±0.75	0.41±0.65
21d	1.59±0.5	0.35±0.57	1.22±0.65	2.58±0.75	0.62±0.79	0.25±0.67
28d	13.32±0.48	0.66±0.68	0.9±0.78	2.03±0.8	1.14±0.55	0.4±0.62
CH60	TLR-7	TLR-3	β-defensin	RIG-1	MDA5	
0.5d	0.82±0.82	0.67±0.72	8.51±0.39	0.41±0.5	0.35±0.76	
1d	0.61±0.86	0.52±0.65	17.75±0.49	0.2±0.85	0.3±0.86	
2d	6.09±0.59	0.67±0.71	93.7±0.42	1.22±0.9	2.23±0.93	
6d	16.91±0.79	1.25±0.64	54.32±0.68	0.27±0.66	0.59±0.67	
8d	0.97±0.68	0.81±0.67	14.49±0.62	0.46±0.57	0.42±0.71	
10d	0.67±0.83	0.84±0.64	0.23±0.54	0.42±0.55	1.04±0.64	
12d	0.47±0.62	0.66±0.53	8.55±0.41	0.01±0.39	0±0.32	
14d	0.56±0.86	0.79±0.59	6.29±0.69	0.22±0.35	0.11±0.34	
21d	1.32±0.74	0.57±0.55	7.91±0.6	0.29±0.67	0.25±0.57	
28d	2.19±0.77	1.33±0.62	5.36±0.54	0.37±0.51	0.25±0.57	

qPCR data of kidney induced by H strain

Table 1 Fold changes of immune related genes induced by DHAV-H strain ($2^{-\Delta\Delta Ct}$)

H	IFN-α	IFN-β	IFN-γ	IL-1β	IL-2	IL-4
0.5d	1.35±0.69	0.09±0.37	0.08±0.29	0.83±0.68	3.74±0.83	5.43±0.6
1d	0.27±0.36	0.12±0.41	0.03±0.45	0.34±0.46	3.65±0.68	10.64±0.38
2d	49.01±0.31	9.59±0.36	4.06±0.46	8.5±0.43	2407.36±0.7	7071.09±0.39
6d	52.53±0.99	4.07±0.19	1.06±0.57	1.44±0.45	176.87±0.82	857.73±0.71
8d	0.79±0.55	12.22±0.26	0.03±0.27	0.38±0.78	5.75±0.36	13.04±0.71
10d	0.64±0.82	0.67±0.43	0.46±0.77	0.8±0.62	0.24±0.78	1.08±0.69
12d	0.38±0.77	0.07±0.23	0.02±0.37	0.81±0.56	4.56±0.77	10.99±0.74
14d	0.18±0.55	0.18±0.31	0.01±0.63	0.27±0.75	6.66±0.63	13.62±0.79
21d	0.06±0.81	0.29±0.37	0.02±0.4	0.06±0.52	4.81±0.42	184.96±0.7
28d	0.34±0.88	4.53±0.4	0.15±0.59	2.63±0.53	55.71±0.67	347.54±0.58
H	IL-6	MHC-I	MHC-II	BAFF	CCL-19	CCL-21
0.5d	1.76±0.82	1.46±0.7	1.85±0.77	1.01±0.55	0.44±0.86	0.4±0.79
1d	1.84±0.83	1.14±0.8	1.53±0.78	1.33±0.75	0.54±0.71	0.41±0.8
2d	32.99±0.55	2.03±0.74	1.34±0.83	4.55±0.71	0.77±0.73	15.2±0.7
6d	52.35±0.7	2.95±0.85	0.91±0.58	8.24±0.48	0.46±0.59	11.26±0.69
8d	3.39±0.48	1±0.83	1.47±0.76	1.23±0.46	0.6±0.62	0.23±0.63
10d	0.42±0.66	0.5±0.55	0.57±0.78	1.62±0.79	0.76±0.66	2.78±0.56
12d	2.23±0.85	0.53±0.74	0.43±0.76	2.96±0.62	0.55±0.63	0.85±0.68
14d	0.6±0.6	0.87±0.48	0.19±0.77	1.3±0.68	0.55±0.61	0.51±0.62
21d	4.2±0.89	0.22±0.73	0.33±0.79	0.96±0.78	0.36±0.72	0.2±0.61
28d	36.25±0.73	0.38±0.85	1.04±0.69	2.67±0.59	0.74±0.58	0.64±0.81
H	TLR-7	TLR-3	β-defensin	RIG-1	MDA5	
0.5d	0.29±0.77	0.67±0.47	12.15±0.32	0.17±0.72	2±0.47	
1d	0.77±0.7	0.52±0.68	26.74±0.55	0.29±0.68	1.78±0.35	
2d	4.6±0.47	1.22±0.73	33.75±0.4	0.44±0.66	4.02±0.31	
6d	2.67±0.54	1.52±0.72	17.04±0.61	0.34±0.71	0.14±0.83	
8d	0.74±0.47	0.6±0.68	2.56±0.44	0.15±0.77	0.69±0.58	
10d	0.96±0.87	0.97±0.67	4.46±0.56	0.18±0.39	0.06±0.46	
12d	0.86±0.61	0.57±0.67	4.4±0.67	0.07±0.63	0.03±0.71	
14d	0.41±0.83	0.72±0.71	7.76±0.8	0.11±0.59	0.01±0.59	
21d	0.94±0.78	0.53±0.7	2.58±0.67	0.14±0.74	0.09±0.72	
28d	5.3±0.81	0.64±0.67	11.34±0.71	0.21±0.79	0.1±0.63	

Data matrix of Pearson Correlation Coefficient caused by CH60 and H strains (Lower-left corner and upper-right corner)

Genes	IFN-α	IFN-β	IFN-γ	IL-1β	IL-2	IL-4	IL-6	MHC-I	MHC-II	BAFF	CCL-19	CCL-21	TLR-7	TLR-3	β-defensin	RIG-1	MDA5
IFN-α		0.42	0.799**	0.674*	0.689*	0.718*	0.803**	0.875**	0.15	0.890**	0.13	0.968**	0.53	0.910**	0.632*	0.830**	0.45
IFN-β	0.988**		0.51	0.53	0.52	0.52	0.40	0.33	0.41	0.26	0.43	0.45	0.46	0.28	0.22	0.44	0.38
IFN-γ	0.719*	0.666*		0.952**	0.980**	0.985**	0.53	0.55	0.21	0.51	0.47	0.911**	0.61	0.657*	0.727*	0.815**	0.760*
IL-1β	0.748*	0.696*	0.999**		0.965**	0.968**	0.54	0.40	0.26	0.42	0.59	0.801**	0.753*	0.51	0.713*	0.751*	0.756*
IL-2	0.891**	0.853**	0.956**	0.967**		0.999**	0.42	0.44	0.23	0.37	0.47	0.818**	0.58	0.50	0.723*	0.746*	0.815**
IL-4	0.874**	0.900**	0.29	0.33	0.56		0.46	0.46	0.22	0.41	0.46	0.840**	0.61	0.53	0.726*	0.766**	0.799**
IL-6	0.749*	0.700*	0.998**	0.999**	0.968**	0.34		.652*	0.12	0.876**	0.21	0.727*	0.816**	0.741*	0.46	0.693*	0.14
MHC-I	0.833**	0.784**	0.967**	0.974**	0.982**	0.47	0.975**		0.40	0.794**	-0.09	0.778**	0.24	0.813**	0.61	0.729*	0.44
MHC-II	0.50	0.47	0.34	0.35	0.42	0.43	0.35	0.47		-0.01	0.09	0.13	0.14	0.01	0.51	0.44	0.658*
BAFF	0.985**	0.971**	0.699*	0.726*	0.874**	0.871**	0.732*	0.809**	0.47		0.07	.799**	0.52	0.867**	0.41	0.63	0.08
CCL-19	-0.04	0.07	-0.12	-0.12	-0.09	0.05	-0.11	-0.10	0.29	-0.08		0.31	0.58	0.22	0.28	0.34	0.26
CCL-21	0.998**	0.987**	0.742*	0.770**	0.906**	.856**	0.770**	0.847**	0.48	0.981**	-0.04		0.58	.889**	0.676*	0.856**	0.55
TLR-7	0.814**	0.776**	0.983**	0.987**	0.986**	0.44	0.991**	0.981**	0.38	0.804**	-0.06	0.831**		0.43	0.49	0.63	0.29
TLR-3	0.24	0.29	0.54	0.53	0.46	-0.02	0.55	0.48	-0.12	0.24	0.31	0.26	0.56		0.43	0.706*	0.22
β-defensin	0.962**	0.956**	0.53	0.56	0.752*	0.948**	0.57	0.693*	0.60	0.953**	-0.01	0.949**	0.649*	0.05		0.885**	0.815**
RIG-1	0.652*	0.706*	0.02	0.06	0.29	0.879**	0.06	0.22	0.35	0.63	0.23	0.62	0.18	-0.05	0.756*		0.689*
MDA5	0.742*	0.790**	0.17	0.21	0.42	0.899**	0.20	0.33	0.35	0.691*	0.26	0.730*	0.30	-0.06	0.803**	0.929**	

**，P< 0.01; *, P<0.05.

References

[1] Adams SC, Xing Z, Li J, Cardona CJ. Immune-related gene expression in response to H11N9 low pathogenic avian influenza virus infection in chicken and Pekin duck peripheral blood mononuclear cells. Molecular immunology 2009;46:1744-1749.
